# Clinically Relevant Interactions between Atypical Antipsychotics and Anti-Infective Agents

**DOI:** 10.3390/ph13120439

**Published:** 2020-12-02

**Authors:** Edoardo Spina, Maria Antonietta Barbieri, Giuseppe Cicala, Jose de Leon

**Affiliations:** 1 Department of Clinical and Experimental Medicine, University of Messina, 98125 Messina, Italy; mbarbieri@unime.it (M.A.B.); gcicala@unime.it (G.C.); 2 Mental Health Research Center at Eastern State Hospital, University of Kentucky, Lexington, KY 40511-1269, USA; jdeleon@uky.edu; 3 Psychiatry and Neurosciences Research Group (CTS-549), Institute of Neurosciences, University of Granada, 18011 Granada, Spain; 4 Biomedical Research Centre in Mental Health Net (CIBERSAM), Santiago Apostol Hospital, University of the Basque Country, 01004 Vitoria, Spain

**Keywords:** antipsychotic agents, antibacterial agents, antibiotics, antitubercular, antifungal agents, antimalarials, antiviral agents, drug interactions, pharmacology, pharmacokinetics

## Abstract

This is a comprehensive review of the literature on drug interactions (DIs) between atypical antipsychotics and anti-infective agents that focuses on those DIs with the potential to be clinically relevant and classifies them as pharmacokinetic (PK) or pharmacodynamic (PD) DIs. PubMed searches were conducted for each of the atypical antipsychotics and most commonly used anti-infective agents (13 atypical antipsychotics by 61 anti-infective agents/classes leading to 793 individual searches). Additional relevant articles were obtained from citations and from prior review articles written by the authors. Based on prior DI articles and our current understanding of PK and PD mechanism, we developed tables with practical recommendations for clinicians for: antibiotic DIs, antitubercular DIs, antifungal DIs, antiviral DIs, and other anti-infective DIs. Another table reflects that in clinical practice, DIs between atypical antipsychotics and anti-infective agents occur in patients also suffering an infection that may also influence the PK and PD mechanisms of both drugs (the atypical antipsychotic and the anti-infective agent(s)). These tables reflect the currently available literature and our current knowledge of the field and will need to be updated as new DI information becomes available.

## 1. Introduction

Drug combinations are increasingly used in the treatment of many conditions and represent an important risk factor for the occurrence of drug interactions (DIs) [1]. A clinically relevant DI occurs when the efficacy or safety of a drug is altered by the concomitant administration of another medication. In a few cases, DIs may prove beneficial, resulting in an increased efficacy or reduced risk of adverse drug reactions (ADRs), and therefore certain drug combinations may be used advantageously in clinical practice. However, more often, DIs are harmful, leading to diminished efficacy or increased toxicity of one or more of the administered medications.

Currently available antipsychotic drugs can be divided into typical or first-generation antipsychotics and atypical or second-generation antipsychotics. In recent years, atypical antipsychotics (amisulpride, aripiprazole, asenapine, brexipiprazole, cariprazine, clozapine, iloperidone, lurasidone, olanzapine, paliperidone, quetiapine, risperidone, and ziprasidone) have progressively replaced older, typical agents due to better safety profiles for reversible extrapyramidal symptoms and tardive dyskinesia [2]. Moreover, new antipsychotics are increasingly prescribed not only for schizophrenia and bipolar disorder, but also for other psychiatric conditions such as adjuvant treatment of major depressive disorder, psychosis and behavioral disorders in dementia, psychosis associated with Parkinson’s disease, resistant obsessive-compulsive disorder, aggressive behavior, and irritability in autism spectrum disorders [2]. Over the past few years, a number of reviews have described the clinically relevant DIs between atypical antipsychotics and various CNS medications including antiepileptics [3] and antidepressants [4].

Anti-infective agents are medications capable of preventing or treating infections. They include a number of widely used classes of drugs such as antibiotics, antituberculosis agents, antifungals, anthelmintics, antimalarials, and antivirals. Many anti-infective medications can lead to DIs [5,6,7].

Considering the frequent co-prescription of anti-infective agents with atypical antipsychotics, it is essential for clinicians to be aware of the potential DIs between them. Previous global reviews of DIs with atypical antipsychotics have also included DIs with anti-infective agents but were not specifically focused on these combinations [8,9]. The purpose of this article was to provide an updated review of clinically significant DIs between atypical antipsychotics and anti-infective agents.

## 2. Methods of Literature Search

After many years of writing articles on antipsychotic DIs [3,4,8], we have figured out that the challenges of finding articles are completely different compared to systematic searches for meta-analysis in which replication by other authors and elimination of low-quality articles with low level of evidence is essential. In the area of DIs, there is so little information that it is not easy to find the articles; therefore, case reports are important, and in their absence, we needed to provide orientation by extrapolating from other antipsychotics with similar pharmacokinetic characteristics. In some cases, we found that even DIs from anti-infective agents with other psychiatric drugs or non-psychiatric drugs were helpful in orienting clinicians when nothing else was available. In summary, we were not trying to exclude articles but to include any information that may help clinicians. Most of the articles that we have included in this review came from our article library on DIs accumulated after writing DI review articles on antipsychotics [3,4,8], which the first and last author keep updating every day as they find articles in their clinical and/or research activities. To be sure that we were not missing any articles from prior searchers or any new articles recently published in September of 2020 we tried to use PubMed searches. PubMed has no MeSH heading for DIs, so we needed to complete many manual searches in a very complex and repetitive way. Each search was done with no time limit from the beginning of PubMed until the day in September that we completed the search. We decided to focus only on articles in English. The searches included 13 atypical antipsychotics and 61 anti-infective agents or classes, leading to 793 individual PubMed searches. Considering erythromycin, for example, we conducted 13 searches by writing in the PubMed search box “erythromycin AND amisulpride”, “erythromycin AND aripiprazole” and the other 11 atypical antipsychotics. Additional relevant DI articles were also obtained from citations of the articles that were retrieved during our search. We also reviewed the Summary of Product Characteristics and Food & Drug Administration (FDA) prescribing information for each atypical antipsychotic and the most commonly used anti-infective agents. Similarly, the Micromedex and the Liverpool DI databases were also searched for any other missing article. In all of these searchers, we only excluded meeting abstracts because they are not easy to obtain. In summary, our search resulted in 34 combinations between an atypical antipsychotic and an anti-infective agent supported by at least one DI study and 52 relevant DI articles from peer-review journals were included. The Introduction and Discussion sections were supported by additional references that helped us to provide a comprehensive review of the topic. The review of DIs in each class of anti-infective agents was summarized in a table with a practical summary of how to manage DIs within that class of anti-infective agents based on the authors’ knowledge at the time of publication of this article. Each table has four columns: anti-infective, atypical antipsychotic, outcome, and actions. When there were studies or case reports we used them to describe outcomes and we added these references in the outcome column. Sometimes there is no published article and the outcome is based on extrapolation from other drugs with similar pharmacological characteristics or pharmacokinetic/pharmacodynamic principles; in this case, no reference is provided. The actions are ordered by letter in an effort to reflect the sequence of decisions; we are just trying to orient clinicians rather than provide strict sequences.

## 3. Basic Mechanisms Involved in DIs between Atypical Antipsychotics and Anti-Infective Agents

The various mechanisms involved in DIs can be categorized as either pharmacokinetic (PK) or pharmacodynamic (PD).

### 3.1. PK DIs

PK DIs consist of modifications in the absorption, distribution, metabolism, or excretion of a drug and/or its metabolite(s) after the addition of another drug. These DIs are associated with a change in plasma concentration of either the drug or its metabolite(s) and are easily verified by therapeutic drug monitoring (TDM).

PK DIs between atypical antipsychotics and anti-infective agents are largely mediated by drug-metabolizing enzymes, in particular, the hepatic cytochrome P450 (CYP) system and, to a lesser extent, the uridine diphosphate glucuronosyltransferase (UGT) system, and by drug transporters in the gut, liver, kidney, and brain such as the P-glycoprotein (P-gp).

The main clinically significant PK DIs between atypical antipsychotics and anti-infective agents occur at a metabolic level and result from enzyme inhibition or induction. Available knowledge of substrates, inhibitors, and inducers of the major drug-metabolizing enzymes has greatly improved the possibility of predicting and eventually avoiding potential DIs [1]. In principle, concomitant treatment with drugs metabolized by the same enzyme or coadministration of a drug with another medication acting as an inhibitor or inducer involves a DI risk. A variety of drug-related (i.e., potency and concentration/dose of the inhibitor/inducer, therapeutic index of the substrate, extent of metabolism of the substrate through the affected enzyme, presence of active metabolites), patient-related (i.e., age, genetic predisposition) and epidemiological factors (i.e., probability of concurrent use) must be taken into account when evaluating the potential occurrence, magnitude and clinical relevance of a metabolic DI [1].

PK parameters of atypical antipsychotics have been described in prior articles [3,4]. In summary, CYP1A2 is the main metabolic pathway for clozapine and olanzapine; CYP3A4 is the main pathway for cariprazine, lurasidone, and quetiapine; CYP2D6 and secondarily CYP3A4 metabolize aripiprazole, brexpiprazole, iloperidone and risperidone; renal elimination is the main way to clear amisulpride and paliperidone; aldehyde oxidase and secondarily CYP3A4 metabolize ziprasidone; and CYP1A2 and UGTs are the main pathways for asenapine. Most atypical antipsychotics appear to be neither inhibitors nor inducers of the major drug-metabolizing enzymes and only asenapine may have weak CYP2D6 inhibitory properties. Therefore, these agents are not expected to give rise to PK DIs with anti-infective agents unless unusual circumstances of saturation are present in which any drug can behave as a competitive inhibitor. On the other hand, many anti-infective drugs may cause metabolically-based DIs because they act as inhibitors or inducers of various drug-metabolizing systems. In this respect, macrolides, fluoroquinolones, isoniazid, azole antimycotics, and antiretrovirals act as strong inhibitors of CYP isoforms including CYP3A4 and CYP1A2 and may therefore impair the elimination of atypical antipsychotics metabolized via these isoforms [10,11,12,13,14]. Conversely, the antitubercular drug rifampin induces the activity of CYP1A2, CYP2C9, CYP2C19, and CYP3A4, as well as UGTs, thereby decreasing the plasma concentrations of a number of newer antipsychotics [15].

PK DIs between atypical antipsychotics and anti-infective agents may also involve drug transporters, in particular P-gp, a multidrug efflux transporter highly expressed in the small intestine, brain, liver, and kidney that acts as a natural defense mechanism against several drugs by limiting their absorption from the gut and penetration into the brain and promoting their elimination in the bile and urine. Among anti-infective drugs, a number of azole antimycotics and antivirals inhibit various drug transporters including P-gp, while rifampin is known to induce the activity of P-gp. There is some evidence suggesting that the atypical antipsychotics risperidone and paliperidone are substrates of P-gp [16,17]. In theory, inhibition of P-gp by certain anti-infective drugs may increase plasma and brain concentrations of these antipsychotics.

### 3.2. PD DIs

PD DIs occur when concomitantly administered medications share the same target sites of actions (i.e., receptor) producing additive, synergistic or antagonistic effects that can enhance or weaken the pharmacological action of either drug without any change in the plasma concentration. While the mechanisms responsible for the therapeutic effect obviously differ between newer antipsychotics and anti-infective drugs, concomitant administration of compounds of these two classes may be associated with ADRs explained by a PD DI.

As a class, antipsychotic medications have the potential to prolong the corrected QT interval (QTc), possibly leading to life-threatening ventricular arrhythmias including Torsades de Pointes (TdP). However, atypical antipsychotics vary markedly in their effects on QTc with ziprasidone and iloperidone associated with the most QTc prolongation and lurasidone, aripiprazole, brexpiprazole, and cariprazine appear to have the least association with QTc prolongation [18,19]. Medications belonging to various antimicrobial classes, including macrolides, fluoroquinolones, azole antimycotics, antiretrovirals, and antimalarials, have been associated with QTc prolongation [20]. Combined use of atypical antipsychotics with anti-infective agents known to prolong the QTc interval may result in additive effects on QTc interval prolongation, potentially leading to cardiac ADRs including TdP and/or sudden death.

Atypical antipsychotics may cause or worsen metabolic abnormalities such as weight gain, hyperglycemia, and hyperlipidemia, specifically the metabolic syndrome, which has been linked to an increased cardiovascular risk [21]. Available evidence indicates that the risk of metabolic disturbances is greatest with clozapine and olanzapine, moderate with risperidone and quetiapine, and least with aripiprazole, ziprasidone, lurasidone, brexpiprazole, and cariprazine [22]. Among anti-infective drugs, some antivirals, namely protease inhibitors, have classically been associated with the development of metabolic changes [23,24]. Potentiation of metabolic effects may occur when these medications are used concomitantly. Moreover, the human immunodeficiency virus (HIV) infection may be also a contributor to metabolic syndrome [25].

Various classes of anti-infective agents can complicate the treatment of patients with prior psychiatric disorders because they have the potential to cause neuropsychiatric ADRs including psychotic symptoms [26,27]. The occurrence of psychotic symptoms induced by anti-infective agents can decrease the benefits of antipsychotic agents.

Rarely some antibiotics have been associated with seizures [28]. Although it is not a well-understood topic, penicillins, cephalosporins, imipenem, and fluoroquinolones may have GABA antagonist properties [28]. All atypical antipsychotics, but clozapine foremost and secondly olanzapine and quetiapine can decrease the seizure threshold [29]. Although this potential PD DI has not been studied, we cannot rule out that in rare patients the combinations of these antibiotics and these atypical antipsychotics might contribute to seizures.

## 4. DIs between Atypical Antipsychotics and Anti-Infective Agents

### 4.1. Antibiotics

Antibiotics are among the most commonly prescribed drugs and many have properties that predispose them to clinically significant DIs. Antibiotics are categorized into different classes based on their mechanism of action, chemical structure, or spectrum of activity. In this section, only classes of antibiotic agents with documented DIs with atypical antipsychotics will be considered in detail.

#### 4.1.1. Macrolides

Macrolide antibiotics, namely erythromycin, clarithromycin, and troleandomycin, are potent inhibitors of CYP3A4 and, to a lesser extent, CYP1A2, and may therefore interfere with the elimination of a number of atypical antipsychotics predominantly metabolized by these enzymes [10].

Controversial findings have been reported concerning the DIs between erythromycin and clozapine. Two case reports have indicated that concomitant treatment with erythromycin resulted in an elevation of plasma levels of clozapine, along with toxic effects such as somnolence, disorientation, dizziness, nausea, and seizures [30,31]. Severe infections release cytokines and inhibit CYPs, including CYP1A2, having the potential to cause a clozapine intoxication; these two case reports did not consider that possibility [32]. A subsequent randomized crossover study in 12 healthy male subjects documented that the PKs of clozapine, administered as a single dose of 12.5 mg, were not significantly modified during coadministration with erythromycin, 1500 mg/day, suggesting a limited involvement of CYP3A4 in the metabolism of clozapine in humans [33]. This study can be criticized because, due to the short duration of the erythromycin phase, steady state was not reached in this study, and doses of clozapine used were lower than those typically used in clinical practice. On the other hand, other studies with potent CYP3A4 inhibitors [34] suggest no relevant effects on clozapine levels. In summary, erythromycin is not likely to cause clozapine intoxication but any severe infection that may be treated with erythromycin is likely to cause a clozapine intoxication.

A clinically significant PK DI may occur between macrolide antibiotics and quetiapine, a second-generation antipsychotic mainly metabolized via CYP3A4. In a PK investigation in Chinese patients with schizophrenia, 19 subjects received multiple doses of quetiapine (400 mg/day) with or without co-administered erythromycin (1500 mg/day) [35]. Concomitant administration with erythromycin was associated with a significant increase in quetiapine maximal plasma concentration (C_max_), area under concentration–time curve (AUC), and terminal-phase elimination half-life time (t_1/2_) by 68, 129, and 92%, respectively, while quetiapine clearance decreased by 52%. These results were explained by inhibition of CYP3A4-mediated metabolism of quetiapine by erythromycin. The potential clinical relevance of this DI is highlighted by the case of a 32-year-old man with schizoaffective disorder and metabolic syndrome who experienced a significant increase in quetiapine plasma concentration following administration of clarithromycin, another macrolide antibiotic and strong CYP3A4 inhibitor. The patient, hospitalized for acute psychotic symptoms, was started with 50 mg/day of quetiapine with a gradual increase in dosage to 700 mg/day over 10 days. Psychotic symptoms disappeared within 3 weeks. On day 28, the patient developed a lower respiratory infection and was treated with oral sultamicillin 750 mg and clarithromycin 500 mg along with his evening dose of quetiapine 400 mg. The following morning, 750 mg sultamicillin, 500 mg clarithromycin, and 300 mg quetiapine were given. Within two hours the patient became somnolent, and plasma quetiapine levels were 827 ng/mL (normal range, 70 to 170 ng/mL). The patient developed severely impaired consciousness and respiratory depression. Quetiapine overdose was suspected and treatment was discontinued. Plasma levels were continually measured over the course of 1 week until complete recovery was achieved [36].

Macrolide antibiotics may prolong the QTc interval [37]. Akathisia, a common antipsychotic ADR, has occasionally been associated with erythromycin and clarithromycin in patients not taking any antipsychotic [38]. Definitive cases of akathisia have been associated when macrolide agents are added to atypical antipsychotics [39].

#### 4.1.2. Fluoroquinolones

Ciprofloxacin, a broad-spectrum fluoroquinolone antimicrobial, is a strong CYP1A2 and a moderate CYP3A4 inhibitor [11] with the potential to inhibit antipsychotics predominantly metabolized by CYP1A2 (clozapine, olanzapine and asenapine).

In a 72-year-old man, the addition of ciprofloxacin treatment (1000 mg/day) on a clozapinemaintenance regimen with a low dose (18.75 mg/day) caused a significant increase in plasma clozapine concentration and suggested an inhibitory effect of ciprofloxacin on CYP1A2 [40]. A formal PK study further investigated this possible DI [41]. In this randomized, double-blind, cross-over study involving 7 schizophrenic inpatients stabilized on clozapine (dose ranging from 150 to 400 mg/day), concomitant administration of ciprofloxacin, 500 mg/day for 7 days, increased mean serum concentrations of clozapine and norclozapine by 29% and 31%, respectively [41]. Interestingly, these two publications involved low doses of either clozapine or ciprofloxacin. On the other hand, a case description by Gex-Fabry et al. revealed the potential for a serious PK interaction, with clozapine levels increasing by a factor of 3.4 from 354 to 1218 ng/mL after the addition of ciprofloxacin 1500 mg/day [42]. This potential DI with ciprofloxacin was formally acknowledged by the FDA in 2005 and reflected in a change in product labeling for clozapine in December 2005. Subsequent case reports [43,44,45,46,47] documented that the PK DI between these two medications could lead to potentially fatal clozapine intoxications.

The possibility of a PK DI between ciprofloxacin and olanzapine mediated by CYP1A2 inhibition was suggested by a case report describing a patient on stable treatment with olanzapine whose serum concentrations were almost doubled after initiation of ciprofloxacin with a dose of 500 mg/day [48].

The potential DI between ciprofloxacin and asenapine, a substrate of CYP1A2 and UGT1A4, was not documented by TDM but suggested by an ADR of sudden-onset dystonia in a patient taking asenapine soon after administration of 1000 mg/day of ciprofloxacin [49].

Two other fluoroquinolones, enoxacin, and norfloxacin, are also clinically relevant inhibitors of CYP1A2 [50] but there is no documentation in the literature of a DI with atypical antipsychotics. On the other hand, levofloxacin is not believed to be a CYP1A2 inhibitor [51] and did not influence clozapine levels in a patient with pneumonia [52].

Fluoroquinolones have been associated with QTc interval prolongation [53]. Letsas et al. [54] reported the case of an elderly patient receiving olanzapine who developed a marked QTc interval prolongation after intravenous administration of ciprofloxacin.

#### 4.1.3. Tetracyclines

Minocycline, a tetracycline antibiotic, has been reported to have beneficial effects on symptoms of schizophrenia when administered as an add-on to antipsychotic therapy [55]. In a study in clozapine-treated schizophrenic patients with persistent symptoms, 52 subjects were randomized to receive minocycline, at a dose of 200 mg/day, or placebo for 10 weeks [56]. Coadministration with minocycline was associated with a statistically significant increase (*p* = 0.033) in clozapine plasma levels as compared to placebo, while no differences in plasma levels of norclozapine were found between the two groups. As minocycline has been found to increase the exposure to theophylline, a substrate for CYP1A2, it was hypothesized that inhibition of CYP1A2 may explain the increase in plasma clozapine concentrations. The article’s research section did not provide data on the effect size of the inhibition but it appears not to be clinically relevant since the discussion stated, “the average increase in clozapine plasma level for the minocycline-treated group was 101.4 ng/mL, approximately a 21% increase from baseline”.

#### 4.1.4. Trimethoprim

Trimethoprim is part of the combined antibiotic trimethoprim-sulfamethoxazole. Trimethoprim is an inhibitor of some renal transporters called organic cation transporters (OCTs) and multidrug and toxin extrusion transporters (MATEs) [57]. A DI study in 30 healthy males by the paliperidone marketer found no effects on paliperidone clearance, suggesting that other renal transporters should be involved in paliperidone clearance [58]. It has been estimated that in normal individuals approximately 38% of paliperidone is excreted by a renal transporter that still needs to be identified [59].

#### 4.1.5. Ampicillin

Csik and Molnar [60] proposed that a case of symptoms of clozapine intoxication during sinusitis was explained by ampicillin inhibition of clozapine metabolism. As ampicillin is not known to be a CYP inhibitor [5], the case was reinterpreted by proposing that the clozapine intoxication was explained by the cytokines released during the sinusitis [32]. The lack of another report describing other cases of DIs in PubMed between clozapine and any other antipsychotic suggests that in effect ampicillin is not a CYP inhibitor. Ampicillin is very frequently used around the world including in patients taking atypical antipsychotics.

A practical summary of managing DIs between antibiotics and atypical antipsychotics is reported in Table 1.

### 4.2. Antitubercular Agents

Rifampin, isoniazid, pyrazinamide, and ethambutol are first-line antitubercular medications which are FDA-approved and indicated for the treatment of mycobacterium tuberculosis. Rifampin and isoniazid are the only antituberculars with DI data with atypical antipsychotics.

#### 4.2.1. Rifampin

The antitubercular agent rifampin is a potent inducer of both the hepatic and intestinal CYPs, including CYP1A2 and CYP3A4, and the P-gp transport system [15]. Thus, it is expected to induce the metabolism of most atypical antipsychotics which are cleared via CYP isoforms and/or are P-gp substrates. Generally, full induction is reached in about 1 week after starting rifampin treatment and the induction dissipates in roughly 2 weeks after discontinuing rifampin [61,62]. The induction is near maximal at a dose of 300 mg/day [61].

Three case reports have documented the possibility of a PK DI between rifampin and clozapine [63,64,65]. The first case reported a 33-year-old male schizophrenia patient stabilized on clozapine therapy (400 mg/day) who developed tuberculosis and was treated with rifampin 600 mg/day [63]. Within 2–3 weeks, his clozapine plasma concentrations decreased approximately six-fold resulting in an exacerbation of psychotic symptoms. A second case described a loss of clozapine efficacy caused by rifampin in a 30-year-old man with schizophrenia [64]. Clozapine therapy for 3 months at 300 mg/day controlled symptoms with some dose-related ADRs (sedation, and hypersalivation). Following diagnosis of tuberculosis, treatment with oral rifampin, 600 mg daily as monotherapy, was started. Two weeks later, the patient no longer complained of sedation and hypersalivation, but his psychotic symptoms worsened. At the end of the month, his psychopathology was as severe as when clozapine was first initiated. The dose of clozapine was increased to 550 mg daily with only mild improvement. Following discontinuation of rifampin after 6 months of therapy, psychotic symptoms improved significantly, while sedation and hypersalivation reappeared within 1 week. Though clozapine concentrations were not reported, fluctuations in symptomatology and ADR were probably explained by an inducing effect of rifampin on clozapine metabolism. In the third case, a 30-year-old female with paranoid schizophrenia had been relatively stable for many years on 100 mg/day of clozapine [65]. Following the introduction of rifampin in a dose of 600 md/day, she experienced an acute and severe psychotic relapse. Her clozapine and norclozapine plasma concentrations were subtherapeutic (less than 50 ng/mL). After discontinuation of rifampin, the clozapine dose was slowly titrated up to 650 mg/day (clozapine levels 400 ng/mL) and over 2–3 months clinical symptomatology gradually improved.

Two formal PK investigations [66,67] evaluated the effect of rifampin on risperidone (metabolized by CYP2D6 and, to a lesser extent, by CYP3A4). In an open, randomized two-phase crossover study involving 10 Thai male volunteers, subjects received oral risperidone 4 mg alone or oral rifampin 600 mg daily for 5 days followed by oral risperidone 4 mg [66]. Concomitant administration of risperidone with rifampin was associated with a significant decrease in the AUC (by 72%; *p* < 0.01), C_max_ (by 50%; *p* < 0.05) when compared with risperidone alone. A randomized, open-label, 2-way crossover study in 10 healthy Korean volunteers found similar results [67]. The pharmacokinetics of a single 1 mg oral dose of risperidone were investigated before and after 7 days with 600 mg rifampin or placebo. Rifampin significantly decreased the AUC of risperidone, 9-hydroxyrisperidone, and the active moiety by 51%, 43%, and 45%, respectively. These two studies provided in vivo evidence of the involvement of CYP3A4 in the metabolism of risperidone but P-gp induction may also be a contributing factor.

Lurasidone is mainly metabolized by CYP3A4. In 20 healthy volunteers, the coadministration of a single dose of lurasidone 40 mg with rifampin 600 mg/day at steady-state (for 8 days) resulted in a decrease in lurasidone C_max_ and AUC by approximately 85% and 82%, respectively, relative to lurasidone administration alone [68].

#### 4.2.2. Isoniazid

Isoniazid is a clinically relevant CYP1A2 inhibitor [13]. A case report described a 65-year-old Caucasian male with a clozapine concentration-dose (C/D) ratio of 0.99 ng/mL per mg/day (and a total C/D ratio of 1.95). The addition of isoniazid increased the C/D (and total 1.95) ratios between 1.73–2.41 (2.73–3.82) while its discontinuation led to normalization of the clozapine C/D ratio to 1.20 (2.30) [69]. According to an in vitro study, isoniazid may be a clinically relevant CYP3A4 inhibitor [70], thus it may be a clinically relevant inhibitor of antipsychotics metabolized by CYP3A4.

A practical summary of managing DIs between antibiotics and atypical antipsychotics is included in Table 2.

### 4.3. Antifungals

Azole antimycotics have the potential to cause clinically significant DIs when co-administered with atypical antipsychotics.

#### Azole Antimycotics

Azole antifungal drugs (namely, fluconazole, itraconazole, ketoconazole, posaconazole, and voriconazole) are used frequently in a clinical setting for prophylaxis or treatment of systemic fungal infections. They are CYP and P-gp inhibitors [12]. Their inhibitory potential varies greatly: itraconazole, ketoconazole, and posaconazole are more potent inhibitors of CYP3A4 than are fluconazole or voriconazole [12]. In addition to CYP3A4, fluconazole and voriconazole are also strong noncompetitive or mixed-type inhibitors of CYP2C9 and CYP2C19 [71].

Ketoconazole has been tested in clinical studies during the development of various atypical antipsychotics that are metabolized by CYP3A4 [9].

In 27 patients treated with quetiapine, concomitant administration of ketoconazole, 400 mg/day, resulted in a 4-fold increase in a mean quetiapine peak plasma concentration [72]. In 12 healthy volunteers receiving 25 mg quetiapine before and after 4 days of treatment with ketoconazole 200 mg daily, concomitant use of ketoconazole increased the mean C_max_ and AUC of quetiapine by 235% and 522%, respectively, and decreased its clearance by 84% [73]. In a study in 10 healthy subjects, coadministration of a single dose of lurasidone 10 mg with ketoconazole 400 mg/day at steady state (for 5 days) caused a 9.3-fold increase in lurasidone AUC, and a 6.8-fold increase in C_max_, as compared to lurasidone alone [68]. These findings are consistent with inhibition of CYP3A4 by ketoconazole.

In an open-label, randomized, crossover study conducted in 10 healthy male volunteers, the PKs of a single oral dose of 2 mg of risperidone were investigated alone or in combination with ketoconazole, 200 mg/day for 3 days [74]. Ketoconazole pretreatment resulted in a significant increase in the AUC of risperidone by 67% (*p* < 0.001) and a decrease in its clearance by 35% (*p* < 0.05).

In a placebo-controlled, randomized, crossover study in 14 healthy subjects, concomitant administration of ziprasidone, given as a single dose of 40 mg, with ketoconazole, 400 mg/day for 2 weeks, was associated with a modest increase in ziprasidone exposure [75]. Mean C_max_ and AUC of ziprasidone increased significantly (*p* < 0.05) by 34% and by 33%, respectively, as compared with placebo.

Two studies in patients with schizophrenia have investigated the possibility of a PK DI between azole antimycotics and clozapine. A double-blind, placebo-controlled, randomized study, performed in 7 schizophrenia patients, documented no significant changes in PK parameters of clozapine during coadministration with itraconazole, 200 mg/day for one week [76]. In a study involving 5 schizophrenia patients, a single clozapine dose of 50 mg was given before and after 7 days of co-administration with ketoconazole 400 mg/day [77]. PK parameters of clozapine and its two major metabolites, norclozapine and clozapine-*N*-oxide, did not change significantly during ketoconazole co-administration. The findings of these two studies indicate that CYP3A4 inhibition may not be clinically significant compared to CYP1A2.

Jung et al. [78] investigated the effect of a treatment with itraconazole, 200 mg/day for a week, on plasma concentrations of risperidone and its active metabolite in 19 schizophrenia patients stabilized on risperidone therapy (2–8 mg/day) in relation to CYP2D6 genotype. Itraconazole increased the mean steady-state plasma concentrations of risperidone active fraction by 71% and 73% in CYP2D6 extensive and poor metabolizers, respectively. As the ratio of risperidone/9-OH-risperidone, an index of CYP2D6 activity was not affected by itraconazole administration, this DI was attributed to inhibition of CYP3A4, an isoform playing a secondary role in the 9-hydroxylation of risperidone. In another PK investigation, coadministration of itraconazole, 100 mg/day for 7 days, to 24 healthy male volunteers increased the C_max_, the AUC, and the half-life of aripiprazole and its main metabolite by 19.4%, 48.0%, and 18.6% and by 18.6%, 38.8%, and 53.4%, respectively [79].

The PK of single doses of lurasidone (20 mg) were investigated in 11 healthy normal-weight volunteers and 13 otherwise healthy obese subjects before and during the administration of posaconazole, 300 mg/day [80]. During posaconazole coadministration, the AUC of lurasidone increased by a factor of 6.2 in normal volunteers and by a factor of 4.9 in obese subjects, reflecting the strong inhibitory effect of posaconazole on CYP3A4 activity.

Azole antifungal agents are known to cause QT interval prolongation [81]. Therefore, concomitant use of newer antipsychotics with azole antimycotics may result in overlapping cardiac toxicity.

Ketoconazole was studied under very well-controlled randomized conditions but eliminating patients with cardiac risk. In the study funded by the ziprasidone marketer, 400 mg/day of ketoconazole was added to 2 atypical antipsychotics [82]. In 31 patients taking 160 mg/day ziprasidone led to a mean QTc increase of 15.9 ms from baseline with almost no change after adding ketoconazole. In 27 patients, taking 750 mg/day of quetiapine led to a mean QTc increase of 5.7 ms from baseline with some increase after adding ketoconazole (value in figure was estimated to be around 8 ms). In the study funded by the iloperidone marketer, 400 mg/day of ketoconazole was added to 3 atypical antipsychotics [83]. Adding ketoconazole in 35 patients taking 160 mg/day ziprasidone led to a mean QTc increase of 15.9 ms from baseline and 6 ms from only ziprasidone. Adding ketoconazole, in 35 patients taking 750 mg/day of quetiapine led to a mean QTc increase of 2.6 ms from baseline. Adding ketoconazole in 39 patients taking 16 mg/day of iloperidone and 20 mg/day of paroxetine the QTc increased as an average 15.7 ms from baseline. Adding ketoconazole in 39 patients taking 24 mg/day of iloperidone and 20 mg/day of paroxetine the QTc increased as an average 19.3 ms compared to baseline. Adding ketoconazole in 35 patients taking 48 mg/day of iloperidone and 20 mg/day of paroxetine the QTc increased as an average 19.5 ms compared to baseline. In summary, in this ketoconazole dose and in these selected patients in those two antipsychotic trials, the effects of ketoconazole on QTc appear to be mainly explained by a PK DI and there were limited effects of the increase of QTc due to PD cardiac effects of ketoconazole.

All azoles had the risk of further QTc prolongation when prescribed with any atypical antipsychotic and this may be potentiated by their PK effects.

A practical summary of managing DIs between antibiotics and atypical antipsychotics is reported in Table 3.

### 4.4. Antivirals

Pharmacological treatment of viral infections has evolved significantly over the last decades with a large number of new agents actually available. The prevalence of some severe viral infections including HIV, hepatitis B, and hepatitis C in patients with severe psychiatric disorders is much higher than in the general population [84]. Many people with psychiatric problems engage in behaviors that increase their risk of infection with blood-borne viruses, including intravenous drug use resulting from co-occurring substance misuse problems and unprotected sex with multiple partners. Atypical antipsychotic drugs are frequently co-prescribed with antiviral agents used to treat these severe infections, possibly resulting in clinically significant DIs.

#### 4.4.1. Antiretrovirals

Psychotic symptoms are relatively common in patients with HIV, so that antipsychotics and antiretroviral agents may be prescribed in combination. Antiretroviral drugs include various classes such as nucleoside/nucleotide reverse transcriptase inhibitors (i.e., zidovudine, lamivudine, abacavir, emtricitabine, tenofovir), non-nucleoside reverse transcriptase inhibitors (i.e., delavirdine, efavirenz, nevirapine, etravirine, rilpivirine), and protease inhibitors (i.e., atazanavir, darunavir, fosamprenavir, indinavir, lopinavir, nelfinavir, ritonavir, saquinavir). Many antiretroviral drugs are prone to PK DIs because they are substrates as well as inhibitors of inducers of several CYP isoforms [14].

All members of the protease inhibitor class are particularly strong inhibitors of CYP3A4, with ritonavir being the most potent. Indeed, this drug is commonly used at low doses in HIV treatment (100–200 mg/d) not for its antiviral activity but rather to take advantage of its inhibition of CYP3A4, which “boosts” the plasma concentrations of co-administered protease inhibitors and thereby allows for once-daily dosing. Ritonavir has a greater potential for DI than other protease inhibitors because it also inhibits CYP2D6. Moreover, ritonavir and indinavir are potent inducers of CYP1A2 and UGT. In addition, protease inhibitors are also substrates and inhibitors of P-gp. Non-nucleoside reverse transcriptase inhibitors also have a variety of effects on CYP isoforms. With regard to this, efavirenz, nevirapine, and etravirine are inducers of CYP3A4, whereas efavirenz and etravirine are inhibitors of CYP2C9 and CYP2C19. Rilpivirine does not substantially affect CYP3A4 and is therefore less commonly involved in DI than other members of this class.

Modern combination antiretroviral therapy consists of at least three antiretroviral agents which may obviously enhance the risk of DIs. Clinically relevant DIs between antiretrovirals and psychotropic drugs including atypical antipsychotics have recently been reviewed [85,86].

A clinically significant DI may occur when risperidone is co-administered with protease inhibitors. A number of case reports have documented the occurrence of ADRs such as extrapyramidal symptoms [87], reversible coma [88], neuroleptic malignant syndrome [89], and late-onset angioedema [90] in patients receiving risperidone associated with ritonavir/indinavir, presumably due to inhibition of risperidone metabolism. As ritonavir is known to be a strong inhibitor of both CYP3A4 and CYP2D6 and indinavir is known to inhibit CYP3A4, concomitant administration of indinavir or ritonavir would be expected to alter metabolism of risperidone.

Quetiapine is also a CYP3A4 substrate and concomitant treatment with ritonavir and other protease inhibitors can increase its serum concentrations. Pollack et al. [91] described two patients who developed weight gain and hyperglycemia (first case) and marked sedation and mental confusion (second case) after the addition of quetiapine to an antiretroviral regimen containing ritonavir and atazanavir. As with ritonavir, atazanavir is also a moderate inhibitor of CYP3A4. While there are no specific recommendations in the quetiapine labeling for dose adjustments with protease inhibitors, if concurrent use is necessary, the quetiapine dose should be reduced to one-sixth of the original dose [86]. Hantson et al. [92] described a case of quetiapine overdose (total 8000 mg ingested) in a 47-year-old female patient concomitantly taking lamivudine (300 mg/day), ritonavir (100 mg/day), atazanavir (350 mg/day), and tenofovir (245 mg/day) for an HIV infection. Major clinical complications consisted of a deep coma and sustained hypotension. The PK data documented a quetiapine half-life of 62 h, which is 10 times the half-life reported in quetiapine’s package insert, and still nearly 3 times the 22-h terminal elimination half-life calculated for a patient following quetiapine overdose (3000 mg) in the absence of CYP3A4 inhibitors [93]. Such a prolonged terminal half-life of quetiapine observed in this case was explained either by CYP3A4 saturation in overdose conditions or by CYP3A4 inhibition by concomitant medications.

Aripiprazole is metabolized by CYP2D6 and CYP3A4. Two case reports are available describing ADRs with the co-administration of aripiprazole and antiretroviral therapy. The first case describes an antiretroviral regimen including darunavir boosted with ritonavir, and concomitant aripiprazole, duloxetine, and buspirone [94], which led to multiple hospital admissions as well as supratherapeutic aripiprazole serum concentrations attributed to a DI between aripiprazole and the antiretroviral therapy. Conversely, in a case of a patient receiving antiretroviral containing a ritonavir-boosted protease inhibitor, the patient experienced lower-than-expected aripiprazole serum levels with clinical worsening after being converted from oral to depot aripiprazole using the manufacturer’s recommended conversion [95]. The patient was ultimately stabilized on double the recommended monthly dose.

Lurasidone is primarily metabolized via CYP3A4. Ritonavir and other protease inhibitors, such as nelfinavir and saquinavir, are specifically mentioned in the lurasidone package insert as examples of strong CYP3A4 inhibitors which are contraindicated with lurasidone. Atazanavir is a moderate CYP3A4 inhibitor and lurasidone dose should be halved when given with atazanavir. Despite these theoretical interactions, there is limited information on these expected DI. Naccarato et al. reported the case of a 63-year-old HIV patient, concomitantly treated with lurasidone and atazanavir, who developed extrapyramidal symptoms associated with elevated serum concentrations of lurasidone. Extrapyramidal adverse effects disappeared following atazanavir discontinuation [96]. One major confounder in this case was the possible contribution of concomitant risperidone to parkinsonian symptoms.

Olanzapine is metabolized via CYP1A2 and UGT1A4. Ritonavir can induce CYP1A2 and UGTs, thus raising concern about the possibility of subtherapeutic olanzapine levels with coadministration. In an open-label crossover study in 14 healthy volunteers, the PKs of a single 10 mg oral dose of olanzapine were investigated before and during coadministration of ritonavir, 600–1000 mg/day [97]. Administration of ritonavir resulted in a significant 53% decrease in olanzapine AUC (*p* < 0.001), in a significant decrease in olanzapine half-life from 32 h to 16 h (*p* < 0.0001), and in a significant increase in olanzapine clearance (*p* < 0.001). The ritonavir doses in this particular study are much higher than in modern practice. Another open-label crossover study, conducted in 20 healthy subjects, investigated the DI between olanzapine and ritonavir, employing a 200 mg/day dose of ritonavir, which more accurately reflects modern-day practice [98]. Increasing the olanzapine dose by 50% (from 10 to 15 mg/day) when co-administered with fosamprenavir 700 mg/ritonavir 200 mg/day (for 16 days) compensated for the induction (of CYP1A2- and UGT-mediated) olanzapine metabolism and resulted in olanzapine exposure that was comparable to when olanzapine 10 mg was administered alone. Based on the results of these studies, a 50% increase in olanzapine dose is warranted when given concomitantly with ritonavir containing antiretroviral regimens.

Being a potent inducer of CYP3A4, efavirenz may decrease plasma concentrations of some newer antipsychotics and reduce their efficacy.

In addition to potential PK DIs, concomitant use of newer antipsychotics with antiretrovirals may be associated with ADR potentiation by PD mechanisms.

Both newer antipsychotics and antiretrovirals, in particular protease inhibitors, are associated with metabolic abnormalities such as weight gain, hyperglycemia, and hyperlipidemia. Concerning protease inhibitors, these ADRs were reported more often with older agents (i.e., ritonavir) than with newer compounds (i.e., darunavir and atazanavir) [23,99,100]. Despite the high rate of HIV and psychiatric comorbidity and the known metabolic effects of atypical antipsychotics and antiretrovirals, the metabolic consequences of this combined treatment have received virtually no coverage in the literature. With regard to this, a retrospective study including 2229 patients with HIV and severe mental illness, examined the effect of concurrent use of atypical antipsychotics and antiretrovirals on metabolic parameters associated with increased cardiovascular risk [101]. Patients treated with both antiretrovirals and atypical antipsychotics were more likely to have higher blood pressure, higher diabetes prevalence, and higher serum triglycerides as compared to those only taking an atypical antipsychotic. General recommendations for the management of overlapping metabolic toxicities in patients receiving both antiretrovirals and newer antipsychotics include evaluating underlying risk, monitoring laboratory parameters, encouraging lifestyle modifications, switching agents if possible or adding additional therapy, communicating among providers, and providing patient education [99].

A potential PD DI that may occur when combining atypical antipsychotics with a number of antiretrovirals is additive QTc prolongation. Among antiretrovirals, the protease inhibitors lopinavir, nelfinavir, ritonavir and saquinavir, and the non-nucleoside reverse transcriptase inhibitors efavirenz and rilpivirine have been associated with QTc prolongation [20,102].

Clozapine has been documented to be effective in HIV patients with refractory schizophrenia. However, its use is associated with the risk of hematologic toxicity resulting in leukopenia, neutropenia, and potentially fatal agranulocytosis. Of the antiretrovirals, zidovudine is known to have myelosuppressive effects [103]. Their combined use should be avoided. While psychosis is a recognized manifestation of HIV, psychotic symptoms may be secondary to certain antiretrovirals including efavirenz, zidovudine, nevirapine, and ganciclovir [104,105,106] and they may theoretically reduce the benefits of concomitantly administered atypical antipsychotics.

#### 4.4.2. Direct-Acting Antivirals

For several years, pegylated interferon-α and ribavirin have been the only available treatment for chronic hepatitis C virus (HCV). Since the development of novel direct-acting antivirals these agents are no longer used. There are three main classes of direct-acting antivirals, depending on the nonstructural protein that target for inhibition: NS3/4A protease inhibitors (boceprevir, telaprevir, simeprevir, grazoprevir, paritaprevir, glecaprevir, voxilaprevir), NS5A inhibitors (ledipasvir, daclatasvir, ombitasvir, elbasvir, velpatasvir, pibrentasvir) and NS5B polymerase inhibitors (sofosbuvir, dasabuvir). These agents are usually given in combinations of two to up to five drugs, thereby enhancing the possibility of DIs.

Each of the available direct-acting antivirals has its own metabolism and presents a different potential for DIs [107]. Moreover, these agents can potentially interact with a variety of psychotropic agents via CYPs and P-gp. Smolders et al. reviewed DIs between the first approved direct-acting antivirals and other concomitant medications including antipsychotics [108]. A recent review by Roncero et al. has specifically assessed the DI potential of each of the currently available direct-acting antivirals with psychotropic drugs [109]. Though these agents are frequently co-prescribed with atypical antipsychotics, no study has investigated the possibility of DIs between agents of these two classes. Based on theoretical knowledge of the PK properties of drugs [108,109], boceprevir, simeprevir, the combination regimen paritaprevir/ombitasvir/ritonavir/dasabuvir and the combination elbasvir/grazoprevir are the most likely direct-acting antivirals to cause DIs with some antipsychotics via the inhibition of CYP3A4 and/or P-gp (paliperidone). On the other hand, no clinically significant DIs are expected when atypical antipsychotics are co-administered with the most commonly used combination regimens including velpatasvir/sofosbuvir and glecaprevir/pibrentasvir.

A practical summary of managing DIs between antibiotics and atypical antipsychotics is included in Table 4.

### 4.5. Other Anti-Infective Agents

Use of the antimalarials chloroquine and hydroxychloroquine and the antiparasitic pentamidine can lead to a prolongation of the QT interval, possibly increasing the risk of TdP and sudden cardiac death [20]. These agents should not be combined with atypical antipsychotics with higher risk for QTc prolongation, such as amisulpride, iloperidone and ziprasidone, and iloperidone.

The possibility of PK DIs caused by these two agents has not been systematically studied but hydroxychloroquine can be a clinically relevant CYP2D6 inhibitor [110] and can increase the serum concentrations of aripiprazole, brexpiprazole, iloperidone, and risperidone. On the other hand, chloroquine does not appear to act as a CYP2D6 inhibitor [111].

A practical summary of managing DIs between antibiotics and atypical antipsychotics is reported in Table 5.

## 5. Contribution of Infections

Using information from Section 4, Table 1, Table 2, Table 3, Table 4 and Table 5 provides a summary of the DI of different classes of anti-infective agents. These tables ignore the fact that patients taking anti-infective agents and atypical antipsychotics frequently have active infections that may also have effects at the PK and PD mechanisms influenced by anti-infective agents and/or atypical antipsychotics. Although it is impossible to review all of these possible PK and PD effects caused by infections, Table 6 tries to provide some practical guidelines when clinicians face acute new problematic symptoms in patients taking anti-infective agents and atypical antipsychotics. They include QTc prolongation, psychosis, seizures, and symptoms compatible with an antipsychotic intoxication. Of those, we only discuss the two most lethal ADRs: QTc prolongation and pneumonia with clozapine intoxication.

Extreme QTc prolongation > 450 ms is usually considered a risk marker for TdP which is potentially lethal. Sudden deaths associated with TdP have an incidence in the range of 1/1000–1/10,000 patients; therefore, most clinicians using antipsychotics may never have seen a case of TdP but, unfortunately, they continue to occur [112]. In the experience of the authors, the problem is that some psychiatrists ignore all or most DIs in their patients and this may not be lethal until the “wrong” patient is treated by them [113]. So using high doses of antipsychotics or combining them with other QTc-prolonging drugs can be non-lethal in many patients, but then cause a sudden death when multiple risk factors are accumulated in a specific patient. Moreover, a very high dose of an antipsychotic can be safe in a prior admission, but then cause a sudden death presumably explained by TdP when other factors are added in the same patient [112]. The recent COVID-19 epidemic in which many patients with serious comorbidity are treated with risky drugs helps to explain the difficulty of extrapolating from QTc prolongation to Torsades de Pointes even in situations of high-risk during infections. In this regard, a recent study included 87 Italian patients treated with 3 anti-infective agents (lopinavir/ritonavir, hydroxychloroquine, and azithromycin) with potential for QTc prolongation and provided a definition of QTc prolongation (an absolute QTc interval ≥ 500 ms or an increase in QTc intervals of ≥60 ms or greater compared with baseline). QTc prolongation was frequent and present in 20 patients [114]. In these 20 patients, 10 had a discontinuation of at least one of these 3 drugs and 3 died. Only 1 case of Torsades de Pointes was identified among 17 surviving patients. Clinicians expecting evidence-based medicine (EBM) approaches in this area must understand that there will never be a well-controlled prospective study that can unequivocally demonstrate that a specific drug combination is safe in a specific patient. These studies cannot accurately replicate the specific characteristics of a specific patient. DIs cannot be understood in the framework of EBM focused on average patients, but rather in the framework of personalized medicine in which each patient is different [115].

Severe infections are usually manifested with signs of generalized inflammation including fever and serum c-reactive protein (CRP) elevations secondary to the cytokines released. The literature is accumulating information that the cytokines can inhibit several CYPs [116]. There is definitive information that inflammation can increase serum clozapine concentrations [32,117]. More limited is the information on olanzapine, which is also metabolized by CYP1A2 [118], and in the antipsychotics dependent on CYP3A4 [119,120]. This peculiar DI, caused by inflammation-released cytokines, may be relevant for clozapine use. Clozapine probably has the narrowest therapeutic index of all atypical antipsychotics [121]. This may explain why in a study of the ADRs reported to the FDA, clozapine ranked as the third most lethal drug with 3277 deaths [122]. In a review of clozapine ADRs reported to an international database by all national drug agencies, pneumonia was associated with 2077 deaths versus 1149 sudden deaths or 550 deaths associated with agranulocytosis [123]. In this database, pneumonia was more closely associated to clozapine than other antipsychotics [124] but, according to a study in a national registry database, the variables associated with treatment-refractoriness may be more important contributors to pneumonia than clozapine [125]. The lethality of pneumonia in clozapine patients is probably due to a combination of pneumonia and the associated clozapine intoxication [126]. Decreasing the clozapine dosage during infections with systemic inflammation may be a good idea [52]. Cases of clozapine intoxication are starting to be described during pneumonia associated with the COVID-19 virus [127].

## 6. Conclusions

After a comprehensive search of the literature, Section 4 describes the limited information published on DIs between atypical antipsychotics and anti-infective agents. The reader should remember that the lack of published studies or case reports does not preclude the occurrence of DIs in their patients. Thus, based on our prior articles of DIs of atypical antipsychotics [3,4,8,29,121] and our current understanding of PK and PD mechanisms, we have developed practical recommendations for clinicians summarized in Table 1 for antibiotic DIs, Table 2 for antitubercular DIs, Table 3 for antifungal DIs, Table 4 for antiviral DIs, and Table 5 for other anti-infective DIs. We have never in prior articles used a table such as Table 6 interpreting DIs in the complexity of clinical practice. DIs between atypical antipsychotics and anti-infective agents do not happen in a laboratory but in a live patient who also has an infection that may influence the PK and PD mechanisms of all administered drugs. These tables reflect the currently available literature and our current knowledge of the field and will need to be updated as new DI information becomes available allowing us to provide better practical recommendations. We remind the reader to look for future updates of this article and others focused on atypical antipsychotic DIs. Hopefully, the reader will agree with us that this article is much better and more practical than our first attempt to write about DIs in atypical antipsychotics 13 years ago [8]. Anyway, with all of its limitations, this is the only published review focused on the DIs between atypical antipsychotics and anti-infective agents. Finally, we encourage researchers to publish new studies on DIs in atypical antipsychotics and anti-infective agents and that, in the absence of these studies, case reports published by clinicians may be the only way to move the field forward.

## Figures and Tables

**Table 1:** Practical summary of managing drug interactions (DIs) between antibiotics and atypical antipsychotics based on the authors’ knowledge at the time of publication of this article.

Antibiotics	Atypical Antipsychotics	Outcome	Actions ^1^
Macrolides: Erythromycin, clarithromycin, and troleandomycin, *are potent inhibitors of CYP3A4*.	Cariprazine, lurasidone, and quetiapine *mainly metabolized by CYP3A4*.	Major ↑ level after adding these macrolides [35,36].	Use another antibiotic when possible; the inhibition is massive and severe ADRs have been described with quetiapine.
	Aripiprazole, brexpiprazole, iloperidone, and risperidone *partly metabolized by CYP3A4*.	↑ level after adding these macrolides. ↓ level after D/C these macrolides.	A. Use another antibiotic when possible. B. No other option: use antipsychotic TDM. C. No other option and no access to TDM: consider antipsychotic dose correction factor of 0.50 during the treatment (based on the effects of CYP34 inhibitors on these antipsychotics).
Macrolides: ↑ QTc *by inhibiting the heart potassium channels*.	High-risk QTc prolongation: amisulpride, iloperidone, ziprasidone	*Additive inhibition of the heart potassium channels*. Greater effects with greater dose or more accurately described with greater serum levels. Remember some macrolides *can increase levels* of iloperidone and risperidone [37].	A. Use another antibiotic or antipsychotic when possible. B. Never use without monitoring QTc.
	Others have intermediate risk.		A. Consider using another antibiotic when possible. B. Monitor QTc. C. Torsades de Pointes is very rare, but the additive risk factors are family history of sudden death; personal history of syncope, arrhythmias or heart conditions; hypokalemia, hypomagnesemia, and co-prescription of other medications that ↑ QTc. Cases are more frequent in females aged > 65 years. D. In the USA, consider the legal risk. Consider the package insert warnings when combining them.
	Lowest risk QTc prolongation: lurasidone, aripiprazole, brexpiprazole and cariprazine.		A. Monitor QTc.
Fluoroquinolones: Ciprofloxacin *is a potent inhibitor of CYP1A2 and moderate CYP3A4*.	Clozapine and olanzapine *are mainly metabolized by CYP1A2*.	↑ level after adding ciprofloxacin. ↓ level after D/C ciprofloxacin [41,42,43,44,45,46,47,48].	A. Use another antibiotic when possible. B. If no other choice, use dose correction factor for clozapine (or olanzapine) of 0.33 and use TDM. C. Consider monitoring QTc.
	Asenapine *is partly metabolized by CYP1A2*.	↑ level after adding ciprofloxacin. ↓ level after D/C ciprofloxacin [49].	A. Use another antibiotic when possible. B. If no other choice, consider decreasing dose and use TDM when available. C. Consider monitoring QTc.
Fluoroquinolones: Enoxacin and norfloxacin *are relevant inhibitors of CYP1A2*.	Clozapine and olanzapine *are mainly metabolized by CYP1A2*. Asenapine *is partly metabolized by CYP1A2*	↑ level after adding antibiotics. ↓ level after D/C antibiotics.	No data. Follow ciprofloxacin recommendation.
Fluoroquinolones: Levofloxacin is believed NOT to be *an inhibitor of CYP1A2*.	Clozapine and olanzapine *are mainly metabolized by CYP1A2*. Asenapine *is partly metabolized by CYP1A2*.	No changes in antipsychotic levels are expected [52].	A. Very limited data. Be careful and monitor patient. B. In clozapine patients use TDM when available.
Fluoroquinolones: ↑ QTc *by inhibiting the heart potassium channels*.	High-risk QTc prolongation: amisulpride, iloperidone, ziprasidone.	*Additive inhibition of the heart potassium channels*. Greater effects with greater dose or more accurately described with greater serum levels. Remember some fluoroquinolones *can increase levels* of clozapine, olanzapine, and asenapine [53,54].	A. Use another antibiotic or antipsychotic when possible. B. Never use without monitoring QTc.
	Other have intermediate risk.		A. Consider using another antibiotic when possible. B. Monitor QTc. C. Torsades de Pointes is very rare, but the additive risk factors are family history of sudden death; personal history of syncope, arrhythmias or heart conditions; hypokalemia, hypomagnesemia, and co-prescription of other medications that ↑ QTc. Cases are more frequent in females aged > 65 years. D. In the USA, consider the legal risk. Consider the package insert warnings when combining them.
	Lowest risk QTc prolongation: lurasidone, aripiprazole, brexpiprazole and cariprazine.		A. Monitor QTc.
Tetracyclines: Minocycline *is a possible mild CYP1A2 inhibitor*.	Clozapine *is mainly metabolized by CYP1A2*.	Mild ↑ level after adding minocycline. Mild ↓ level after D/C minocycline [56].	A. Limited available data suggest that inhibitory effects are not clinically relevant. B. Consider TDM.

ADR: adverse drug reaction; D/C: discontinuing; DI: drug interaction; QTc: corrected QT interval; TDM: therapeutic drug monitoring; ↑: increased; ↓: decreased. *Italics are used in the text to reflect pharmacokinetic or pharmacodynamic mechanisms*. ^1^ A correction factor is used to modify dosing to account for DI. For example, a correction factor of 0.5 indicates that the substrate dose should be halved. The recommendations are based on the limited information available.

**Table 2:** Practical summary of managing DIs between antitubercular agents and atypical antipsychotics based on the authors’ knowledge at the time of publication of this article.

Antitubercular Agents	Atypical Antipsychotics	Outcome	Actions ^1^
Rifampin *is an inducer of multiple metabolic enzymes and transporters*.	Clozapine and olanzapine are *mainly metabolized by CYP1A2*.	↓ level 1 week after adding rifampin. ↑ level 2 weeks after D/C rifampin [63,64,65,66,67,68].	A. Consider another antitubercular agent or atypical antipsychotic when possible. Amisulpride and ziprasidone may be the least influenced by induction. B. No other option: use antipsychotic TDM. C. No other option and no access to TDM we can only provide recommendations based on the effects of potent inducers on these antipsychotics by using dose correction factors of: 2 (for clozapine, olanzapine, aripiprazole, brexpiprazole, iloperidone, or risperidone) and 3 (for paliperidone). D. We cannot provide any dose correction factor for asenapine and access to its TDM is limited. E. We do not recommend combining rifampin with cariprazine, lurasidone, or quetiapine even with access to TDM since, based on the effects of potent inducers such as carbamazepine on these antipsychotics, the needed correction factor may be 5 or even higher.
	Aripiprazole, brexpiprazole, iloperidone and risperidone *are partly metabolized by CYP3A4*.		
	Cariprazine, lurasidone and quetiapine are *mainly metabolized by CYP3A4*.		
	During induction, paliperidone *may be mainly metabolized by CYP3A4.Pg-p induction may also decrease paliperidone effects*.		
Isoniazid *is an inhibitor of CYP1A2 and CYP3A4*.	Clozapine and olanzapine are *mainly metabolized by CYP1A2*.	↑ level after adding isoniazid. ↓ level after D/C isoniazid [69].	A. Consider another antituberculosis agent or atypical antipsychotic when possible. Amisulpride, paliperidone and ziprasidone may be the least influenced by inhibition. B. No other option: use antipsychotic TDM. C. No other option and no access to TDM: we can only provide recommendations based on the effects of potent inhibitors on these antipsychotics by using dose correction factors of: 0.5 (for clozapine, olanzapine, aripiprazole, brexpiprazole, iloperidone, and risperidone). D. We cannot provide any dose correction factor for asenapine and access to its TDM is limited. E. We do not recommend combining isoniazid with cariprazine, lurasidone or quetiapine unless clinician has expertise in their TDM.
	Aripiprazole, brexpiprazole, iloperidone, and risperidone *are partly metabolized by CYP3A4*.		
	Cariprazine, lurasidone, and quetiapine are *mainly metabolized by CYP3A4*.		
	Asenapine *is partly metabolized by CYP1A2*.		

D/C: discontinuing; DI: drug interaction; TDM: therapeutic drug monitoring; ↑: increased; ↓: decreased. *Italics are used in the text to reflect pharmacokinetic or pharmacodynamic mechanisms*. ^1^ A correction factor is used to modify dosing to account for DI. For example, a correction factor of 0.5 indicates that the substrate dose should be halved. The recommendations are based on the limited information available.

**Table 3:** Practical summary of managing DIs between antifungals and atypical antipsychotics based on the authors’ knowledge at the time of publication of this article.

Antifungals	Atypical Antipsychotics	Outcome	Actions
Itraconazole, ketoconazole, and posaconazole *are potent inhibitors of CYP3A4* and they prolong QTc *by inhibiting the heart potassium channels*.	Cariprazine, lurasidone, and quetiapine are *mainly metabolized by CYP3A4*.	↑ level of CYP3A4 antipsychotics after adding azole. ↓ level of CYP3A4 antipsychotics after D/C azole. Additive effects on QTc prolongation [72,73,74,75,76,77,78,79,80,81,82,83].	A. Do not use combinations of these azoles with atypical antipsychotics metabolized by CYP3A4 (cariprazine, lurasidone, quetiapine, aripiprazole, brexpiprazole, iloperidone, and risperidone). B. Do not use combinations of these azoles with atypical antipsychotics with high risk of QTc prolongation (amisulpride, iloperidone, and ziprasidone). C. Adding these azoles to clozapine, olanzapine, and paliperidone may have minimal inhibitory effects on their metabolism but additive effects on QTc prolongation should be expected. Monitoring QTc is recommended.
	Aripiprazole, brexpiprazole, iloperidone, and risperidone *are partly metabolized by CYP3A4*.		
	Clozapine and olanzapine are *mainly metabolized by CYP1A2*.		
	Asenapine *is partly metabolized by CYP1A2*.		
	*Inhibition of metabolism* of amisulpride may be minimal and of ziprasidone may be small but they cause greater prolongation of QTc interval *by inhibiting the heart potassium channels*.		
Fluconazole and voriconazole *appear to be mild to moderate CYP3A4 inhibitors* but they prolong QTc *by inhibiting the heart potassium channels*.	High-risk QTc prolongation: amisulpride, iloperidone, ziprasidone.	Additive effects on QTc prolongation [81].	A. Use another antifungal or antipsychotic when possible. B. Never use without monitoring QTc.
	Others have intermediate risk. More risk for those depending on CYP3A4 for their metabolism (quetiapine and risperidone).		A. Consider using another antifungal when possible. B. Monitor QTc. B. Monitor TDM for quetiapine and risperidone. D. Torsades de Pointes is very rare, but the additive risk factors are family history of sudden death; personal history of syncope, arrhythmias, or heart conditions; hypokalemia, hypomagnesemia, and co-prescription of other medications that ↑ QTc. Cases are more frequent in females aged > 65 years. E. In the USA, consider the legal risk. Consider the package insert warnings when combining them.
	Lowest risk QTc prolongation: lurasidone, aripiprazole, brexpiprazole and cariprazine.		A. Monitor QTc.

D/C: Discontinuing; DI: drug interaction; QTc: corrected QT interval; TDM: therapeutic drug monitoring; ↑: increased; ↓: decreased. *Italics are used in the text to reflect pharmacokinetic or pharmacodynamic mechanisms*.

**Table 4:** Practical summary of managing DI between antivirals and atypical antipsychotics based on the authors’ knowledge at the time of publication of this article.

Antivirals	Atypical Antipsychotics	Outcome	Actions
Antiretrovirals: Ritonavir *is a potent inhibitor of CYP3A4, CYP2D6, and P-gp and an inducer of CYP12 and UGT*. It prolongs QTc *by inhibiting the heart potassium channels*.	Cariprazine, lurasidone, and quetiapine are *mainly metabolized by CYP3A4*. Aripiprazole, brexpiprazole, iloperidone, and risperidone *are partly metabolized by CYP3A4*.	↑ level of these antipsychotics after adding ritonavir. ↓ level of these antipsychotics after D/C ritonavir. Additive effects on QTc prolongation [20,87,88,89,90,91,92,93,94,95,96,102].	A. Do not combine ritonavir with these antipsychotics. Serious ADRs have been described. B. If you decide to take the risk of combining them use antipsychotic TDM and QTc monitoring.
	Clozapine and olanzapine are *mainly metabolized by CYP1A2*.	↓ level after adding ritonavir. ↑ level after D/C ritonavir. Additive effects on QTc prolongation [20,97,98,102].	A. Consider alternatives. B. Use TDM for better dosing of clozapine and olanzapine. C. Monitor QTc.
	Asenapine *is metabolized by UGT and CYP1A2*.	Likely ↓ level after adding ritonavir. Likely ↑ level after D/C ritonavir. Additive effects on QTc prolongation [20,102].	A. Do not combine ritonavir with asenapine. There is no data to provide recommendations.
Antiretrovirals: Indinavir *is an inducer of CYP12 and UGT*. It prolongs QTc *by inhibiting the heart potassium channels*.	Clozapine and olanzapine are *mainly metabolized by CYP1A2*.	↓ level after adding indinavir. ↑ level after D/C indinavir. Additive effects on QTc prolongation.	A. Consider alternatives. B. Use TDM for better dosing clozapine and olanzapine. C. Monitor QTc.
	Asenapine *is metabolized by UGT and CYP1A2*.	Likely ↓ level after adding indinavir. Likely ↑ level after D/C indinavir. Additive effects on QTc prolongation.	A. Do not combine indinavir with asenapine. There is no data to provide recommendations.
Antiretrovirals: Efavirenz, nevirapine, and etravirine *are CYP3A4 inducers*. They prolong QTc *by inhibiting the heart potassium channels*.	Cariprazine, lurasidone, and quetiapine are *mainly metabolized by CYP3A4*. Aripiprazole, brexpiprazole, and risperidone *are partly metabolized by CYP3A4*.	↓ level after adding antivirals. ↑ level after D/C antivirals. Additive effects on QTc prolongation [20,102].	A. Consider alternatives. B. Use TDM for better dosing these antipsychotics. C. Monitor QTc.
	High-risk QTc prolongation: amisulpride, iloperidone, ziprasidone.	Additive effects on QTc prolongation. ↓ level of iloperidone after adding antivirals. ↑ level of iloperidone after D/C antivirals [20,102].	A. Do not combine.
Antiretrovirals: Nelfinavir and saquinavir *are strong inhibitors of CYP3A4* Atazanavir *is a moderate inhibitor of CYP3A4*. They prolong QTc *by inhibiting the heart potassium channels*.	Cariprazine, lurasidone, and quetiapine are *mainly metabolized by CYP3A4*. Aripiprazole, brexpiprazole, and risperidone *are partly metabolized by CYP3A4*.	↑ level after adding antivirals. ↓ level after D/C antivirals. Additive effects on QTc prolongation [20,102].	A. Consider alternatives. B. Use TDM for better dosing of these antipsychotics. C. Monitor QTc.
	High-risk QTc prolongation: amisulpride, iloperidone, ziprasidone.	Additive effects on QTc prolongation [20,102]. ↑ level of iloperidone after adding antiviral. ↓ level of iloperidone after D/C antiviral.	A. Do not combine.
Antiretrovirals: Zidovudine has risk of bone marrow suppression	Clozapine can cause agranulocytosis *possibly due to immunological mechanisms*.	Additive effects cannot be ruled out.	A. Do not combine.
Direct-acting antivirals: Boceprevir, simeprevir, the combination paritaprevir/ombitasvir/ritonavir/dasabuvir, and the combination of elbasvir/grazoprevir *are CYP3A4 and/or P-gp inhibitors*.	Cariprazine, lurasidone and quetiapine are *mainly metabolized by CYP3A4*. Aripiprazole, brexpiprazole, iloperidone, and risperidone *are partly metabolized by CYP3A4*.	↑ level after adding antivirals. ↓ level after D/C antivirals [108,109].	A. Consider alternatives. B. Use TDM for better dosing these antipsychotics.

ADR: adverse drug reaction; D/C: discontinuing; DI: drug interaction; QTc: corrected QT interval; TDM: therapeutic drug monitoring; ↑: increased; ↓: decreased. *Italics are used in the text to reflect pharmacokinetic or pharmacodynamic mechanisms*.

**Table 5:** Practical summary of managing DIs between other anti-infective agents and atypical antipsychotics based on the authors’ knowledge at the time of publication of this article.

Other Anti-Infective Agents	Atypical Antipsychotics	Outcome	Actions ^1^
Hydroxychloroquine *is a CYP2D6 inhibitor*. It prolongs QTc *by inhibiting the heart potassium channels*.	Aripiprazole, brexpiprazole, and risperidone *are partly metabolized by CYP2D6*.	↑ level of these antipsychotics after adding hydroxychloroquine. ↓ level of these antipsychotics after D/C hydroxychloroquine. Additive effects on QTc prolongation [20].	A. Consider alternatives. B. Use TDM for better dosing these antipsychotics. C. In absence of TDM considering a dose correction of 0.5 for these antipsychotics. D. Monitor QTc.
	High risk QTc prolongation: amisulpride, iloperidone, ziprasidone *by inhibiting the heart potassium channels*.	Additive effects on QTc prolongation [20]. ↑ level of iloperidone after adding hydroxychloroquine. ↓ level of iloperidone after D/C hydroxychloroquine.	A. Do not combine.
Chloroquine prolongs QTc *by inhibiting the heart potassium channels*. It may be more prone to cause psychiatric symptoms than hydroxychloroquine.	High risk QTc prolongation: amisulpride, iloperidone, ziprasidone *by inhibiting the heart potassium channels*.	Additive effects on QTc prolongation [20].	A. Do not combine.

D/C: Discontinuing; DI: drug interaction; QTc: corrected QT interval; TDM: therapeutic drug monitoring; ↑: increased; ↓: decreased. *Italics are used in the text to reflect pharmacokinetic or pharmacodynamic mechanisms*. ^1^ A correction factor is used to modify dosing to account for DI. For example, a correction factor of 0.5 indicates that the substrate dose should be halved. The recommendations are based on the limited information available.

**Table 6:** New symptoms during infection when patients are co-prescribed anti-infective agents and atypical antipsychotics.

Situation	Anti-Infective Agents	Atypical Antipsychotics	Actions
During infection: ↑ QTc Consider direct effects of infection in heart.	Risk of ↑ QTc: macrolides, fluoroquinolones, azole antimycotics, some antivirals, and antimalarials *by inhibiting the heart potassium channels*.	All can cause ↑ QTc but highest risk for amisulpride, iloperidone, and ziprasidone *by inhibiting the heart potassium channels*.	A. Avoid using antipsychotics with highest risk. B. Be vigilant even when only one risky drug is present. Consider the need for QTc monitoring. C. Torsades de Pointes is very rare, but the additive risk factors are family history of sudden death; personal history of syncope, arrhythmias, or heart conditions; hypokalemia, hypomagnesemia, and co-prescription of other medications that ↑ QTc. Cases are more frequent in females aged > 65 years. D. In the USA, consider the legal risk. Consider the package insert warnings when combining them.
During infection: exacerbation of psychosis in patients with underlying illness or appearance in patients who never presented psychosis. Consider brain effects of infection.	Some anti-infective agents have been associated as causal agents of psychosis.	All APs have antipsychotic efficacy *by inhibiting D_2_ receptors (antagonists or partial agonists) at basal ganglia and/or cortex*. Occasionally psychotic exacerbations have been suggested by aripiprazole case reports *by being a D2 partial agonist at basal ganglia and/or cortex*.	A. Extremely complex situation. Consider all possible causal factors. B. Involve other medical specialties in diagnosis. C. Psychiatrist needs to carefully review PK/PD DIs of all drugs, one by one, and timing of the psychosis; help other medical specialties to think clearly. D. If possible, change only one variable at a time.
Seizures during infection. Consider brain effects of infection.	Penicillins, cephalosporins, imipenem, and fluoroquinolones *may have GABA antagonist properties*.	*By ↓ seizure threshold*: Highest risk: clozapine. 2nd riskiest: olanzapine and quetiapine. 3rd riskiest: rest of atypical antipsychotics.	A. Extremely complex situation. Consider all possible causal factors. B. Involve neurologist in diagnosis. C. Psychiatrist needs to carefully review PK/PD DIs of all drugs, one by one, and timing of the seizure; help other medical specialties to think clearly. D. If possible, change only one variable at a time.
During infection symptoms compatible with clozapine intoxication (including sedation, hypersalivation, myoclonus, or seizure).	Never use ciprofloxacin, enoxacin, and norfloxacin which *are relevant inhibitors of CYP1A2*.	Presence of fever or CRP elevations are signs that cytokine release *may inhibit CYP1A2*.	A. Tell patient and/or family to call psychiatrist when fever or sign of infection occurs. B. If fever and/or CRP elevation develop, cut clozapine dose in half unless clozapine TDM is immediately available. C. Once clozapine intoxication occurs, cut clozapine to one-third of the dose or stop completely. D. If possible, monitor clozapine TDM. E. Do not go back to prior clozapine dose until CRP is normal.
During infection symptoms compatible with olanzapine intoxication (including sedation).	Never use ciprofloxacin, enoxacin, and norfloxacin which *are relevant inhibitors of CYP1A2*.	Presence of fever or CRP elevations are signs that cytokine release *may inhibit CYP1A2*. Although not well-studied, elevations of serum olanzapine concentrations may be lower than in clozapine.	A. If fever and/or CRP elevation develop and signs of olanzapine intoxication are present, consider cutting olanzapine dose in half unless olanzapine TDM is immediately available. B. If possible, monitor olanzapine TDM. C. Do not go back to prior olanzapine dose until CRP is normal.
During infection symptoms compatible with antipsychotic intoxication and patient on antipsychotic dependent on CYP3A4. Cariprazine, lurasidone and quetiapine are *mainly metabolized by CYP3A4*. Aripiprazole, brexpiprazole, iloperidone and risperidone *are partly metabolized by CYP3A4*.	Never use erythromycin, clarithromycin, and troleandomycin which *are relevant inhibitors of CYP3A4*.	Presence of fever or CRP elevations are signs that cytokine release *may inhibit CYP3A4*. Although not well-studied, elevations of serum concentrations of these antipsychotics may be lower than in clozapine.	A. If fever and/or CRP elevation develop and signs of antipsychotic intoxication are present, consider decreasing the dose unless TDM is immediately available. B. If possible, monitor TDM. C. Do not go back to prior antipsychotic dose until CRP is normal.

CRP: c-reactive protein; D/C: Discontinuing; DI: drug interaction; PK: pharmacokinetic; PD: pharmacodynamic; QTc: corrected QT interval; TDM: therapeutic drug monitoring; ↑: increased; ↓: decreased. *Italics are used in the text to reflect pharmacokinetic or pharmacodynamic mechanisms*.
